# Looking into the Market Behaviors through the Lens of Correlations and Eigenvalues: An Investigation on the Chinese and US Markets Using RMT

**DOI:** 10.3390/e25101460

**Published:** 2023-10-18

**Authors:** Yong Tang, Jason Xiong, Zhitao Cheng, Yan Zhuang, Kunqi Li, Jingcong Xie, Yicheng Zhang

**Affiliations:** 1 School of Computer Science and Engineering, University of Electronic Science and Technology of China, Chengdu 610054, China; tukouacademic@gmail.com; 2 Department of Physics, University of Fribourg, Chemin du Musée 3, 1700 Fribourg, Switzerland; yi-cheng.zhang@unifr.ch; 3 Walker College of Business, Appalachian State University, Boone, NC 28608, USA; xiongjj@appstate.edu; 4 School of Mathematical Sciences, University of Electronic Science and Technology of China, Chengdu 610054, China; 2020110801009@std.uestc.edu.cn; 5 Department of Electrical and Computer Engineering, State University of New York at Stony Brook, Stony Brook, NY 11794, USA; kunqi.li@stonybrook.edu; 6 Terry College of Business, University of Georgia, Athens, GA 30602, USA; jx32164@uga.edu

**Keywords:** financial big data, stock market modeling, random matrix theory, eigenvalue analysis

## Abstract

This research systematically analyzes the behaviors of correlations among stock prices and the eigenvalues for correlation matrices by utilizing random matrix theory (RMT) for Chinese and US stock markets. Results suggest that most eigenvalues of both markets fall within the predicted distribution intervals by RMT, whereas some larger eigenvalues fall beyond the noises and carry market information. The largest eigenvalue represents the market and is a good indicator for averaged correlations. Further, the average largest eigenvalue shows similar movement with the index for both markets. The analysis demonstrates the fraction of eigenvalues falling beyond the predicted interval, pinpointing major market switching points. It has identified that the average of eigenvector components corresponds to the largest eigenvalue switch with the market itself. The investigation on the second largest eigenvalue and its eigenvector suggests that the Chinese market is dominated by four industries whereas the US market contains three leading industries. The study later investigates how it changes before and after a market crash, revealing that the two markets behave differently, and a major market structure change is observed in the Chinese market but not in the US market. The results shed new light on mining hidden information from stock market data.

## 1. Introduction

Thanks to the availability of financial data in a wide range of frequencies from tick to daily, it is possible to apply data mining and knowledge discovery methods beyond traditional finance but from data science, network analysis, and even physics, etc. The asset prices in the markets result from complicated dynamics of spreading and reacting to market signals and information. The market structures are embedded in the price movements, which are normally correlated with each other. As a starting point for the underlying cornerstones of finance theories like modern portfolio theory (MPT) [1] and capital asset pricing model [2], the correlation information of assets prices is always at heart for theoretical studies and finance industrial practices in portfolio management and risk management, etc.

For a portfolio of *N* stocks, we need a correlation matrix with N×N elements to describe the pairwise relationships. With the increase of *N*, the number of possible relationships snowballs, making it difficult and challenging to calculate or analyze. To extract the hidden structure and essential information, it is necessary to simplify the network by filtering the less important elements to make it feasible to analyze portfolios even with a very large *N*. In the past few years, we see some methods have been introduced to simplify the stock matrices. To study the correlation behaviors of the financial markets, a correlation matrix is constructed from the price time series before we apply methods and techniques such as principal component analysis [3,4,5,6], multidimensional scaling [7], factor analysis [4], minimum spanning tree [8,9], hierarchical clustering [8,10,11], and singular value decomposition [12].

Simplification of the correlation matrix requires validation, which statistically validates the matrix and keeps those validated elements to achieve a simple matrix with fewer noises and ease of analysis. The validations provide statistical confidence in the results or insights extracted from the validated matrices. The underlying idea of design validation is to compare the empirical matrices with random ones generated from the same distributions, random shuffles, or statistical tests with which the null hypothesis is set up to be tested with empirical data. Any deviations from these *benchmarks* are considered noises and should be filtered. Similarly, given an empirical correlations matrix (and the derived distance matrices for the networks), we can consider a random matrix with the same size. A null hypothesis can be introduced to test the statistical validation of each element of the original empirical matrix by comparing the distributions. The basic idea is that any deviations from the random distribution are believed as validated with genuine information from the system. In contrast, those falling within the random distribution are pure random noises that contain no system information.

Specifically, in this study, based on a dataset covering nine years of stock prices, we systematically investigate the stock markets of China and the US using random matrix theory (RMT) to study and compare the correlation properties and the dynamics of eigenvalues and eigenvectors. The findings revealed that the two stock markets are both similar and different in many ways. The results add new insights into market behaviors with implications for finance applications such as portfolio management and optimization, market risk monitoring, and trading strategy design. Meanwhile, this study also serves as a framework for data mining and knowledge in financial big data using RMT.

This work is organized as follows. First, we review the literature in Section 2. The methodology is introduced in Section 3. In Section 4 we describe the dataset of two markets and the properties of correlation matrices. Using RMT, in Section 5, the properties and behaviors of eigenvalues and eigenvectors are analyzed with an investigation of a market switch study. Finally, Section 6 presents conclusions, discussions, and limitations.

## 2. Literature Review

In this section, we introduce literature from three aspects. First, RMT and its applications are introduced in Section 2.1, representative studies of applying RMT in analyzing financial markets are described in Section 2.2, as well as recent studies focusing on comparing different stock markets, especially the US and Chinese markets are discussed in Section 2.3.

### 2.1. RMT and Its Applications

Originating in mathematical physics, RMT was first introduced by physicists to study nuclear activities back in the 1950s [13]. Eugene Wigner used RMT to model the excited states of nuclei in reactions which was hard to obtain by using traditional methods. Instead, he proposed to analyze the eigenvalues and their spacing of a random matrix [14]. The basic idea is to analyze the statistical properties of eigenvalues of the random counterpart whereas it is practically impossible to analyze the individual eigenvalues of the original complex system. RMT provides a powerful toolbox to reveal properties of matrices whose elements are sampled from randomness, usually based on certain probability distributions. Soon, RMT was proved an efficient tool for many challenges in physics and beyond. Before long, RMT attracted significant interest from scholars in various fields with wide-spreading applications like physics, mathematics, biology, engineering, computer science, and social science. After decades of development, RMT has become an important research field with rich theoretical implications and real applications in a variety of disciplines, such as spectrum analysis and filter in information processing, signal detection and channel estimation in wireless communication, data analysis in high dimensional space, and optimization in machine learning. Interested readers should refer to works by Potters and Tao for details on theories and applications of RMT [15,16].

### 2.2. Applying RMT Approaches in Financial Markets

In finance, RMT was first introduced into the study of financial markets by [17], and more recently, there are significant advances in applying RMT in finance studies and applications [18,19,20]. In one study, RMT is applied to analyze stock market behaviors [21]. In another study, the world stock market is analyzed with RMT [22]. Recent works also investigated various financial markets using RMT [23,24,25].

#### 2.2.1. RMT in Financial Correlation Analysis

Rooted in the correlation analysis, RMT offers a new look into the structures and behaviors of the financial markets. Applying RMT to financial markets is closely related to the analysis of correlation matrices and network structures [26,27,28,29,30]. The market is full of noises, and the useful information in correlation matrices built from price data might be covered by the noises and make correlation analysis less meaningful [31]. To quantify the validations of correlations, recently, there are many works applying RMT into the studies of the correlation matrices of financial markets [17,31,32,33,34,35,36,37]. Recently, there is an emergence of research using RMT in financial markets to filter noises and reveal embedded market properties. The cross-correlations of stock prices are studied using RMT to identify correlated relationships [38]. Furthermore, free random variables are applied in RMT analysis in financial time series [39]. RMT has also been applied to return estimation and asset allocation in Markowitz mean-variance optimization [40].

#### 2.2.2. RMT in Eigenvalue Analysis

RMT provides a powerful tool for eigenvalue analysis in financial markets. Using time-shifted series, the lagged correlation matrices are studied from the RMT approach to compute eigenvalue density and identify deviations [41]. It has been verified that the largest eigenvalue $\lambda_{max}$ is a good estimator of the average correlation of the correlation matrices constructed from a sliding window approach [42]. The same results are also reported, revealing that the average correlation co-moves with the largest eigenvalue for the component stocks of S&P500 [36]. For normalized eigenvectors, the value of $S_{ij}$ ranges from 0 to 1. In other words, the two eigenvectors change from orthogonal to exactly the same. One study reports that the effect of noises on the risk becomes insignificant in measure of the fixed portfolio while remaining important for an optimized portfolio for small values of NNLL [31]. This indicates that the correlation matrix can still be valid in traditional risk management and portfolio optimization; noises cover even most information. Using simulation methods, many correlation matrix filtering approaches are tested, and the approaches based on random matrix theory are found to perform consistently well in all cases [43]. The eigenvalue distribution of the emerging stock market is different from developed markets though correlation distributions and other properties are similar. Methods based on clustering for portfolio optimization and effective size determination are proposed. The results are found to be improved compared to RMT approaches [37], which indicates that RMT might be further combined with other methods in filtering matrix and optimizing a portfolio [27].

It was found that the average of correlations in the correlation matrix can be well estimated from the largest eigenvalue as

(1) λmaxλmaxNN∼Cij.

Following the RMT approach, the largest eigenvalues are found to be responsible for the market mode. By removing this, the correlation matrix is cleaned to reveal the topological structures [28]. The details of the residual noise part for a market are studied, revealing that the noise band is composed of more sub-bands [11]. Using RMT, the Chinese stock market is studied [18], a similar anti-correlation relationship between sub-sectors is studied [44], and the results show that the prominent sector structure exists. The distribution of eigenvalues also reveals that the market is likely to be influenced by the Chinese government’s global financial crisis and policies. In a further study on the sub-sectors of a stock market, local interaction structures are found to change during financial crises [19]. The sign information of components in eigenvectors is again used to detect the sub-sector anti-correlations [45]. Focusing on how the credit market and stock market behave before and after a financial crisis, RMT is applied and finds that the largest eigenvalue of the credit market precedes that of the stock market [46]; this indicates that the pattern changes of eigenvalues have potential implications in the understanding of interrelationships between different markets. Market contagion is also investigated from financial network analysis and, naturally, RMT. Market contagion is an important indicator of market stability. By looking into the structural changes in networks and properties revealed by RMT, one can identify and predict the market contagion and thus major market switches [47,48,49,50,51].

#### 2.2.3. RMT in Eigenvalue Distributions

Since the introduction of RMT into the study of financial markets, much literature investigated different markets. An earlier study points out that the lower bound is positive and no eigenvalues fall between 0 and $\lambda_{min}$ also vanish above $\lambda_{max}$ [17]. Since the empirical values of *N* and *L* are limited far from *∞*, the edges are blurred with some eigenvalues falling beyond the bounds [32]. The distribution of the spacings of eigenvalues $s\equiv \lambda_{i+1}-\lambda_{i}$ are found to agree with a Wigner distribution of the energy spacing levels [34]. This provides evidence indicating that the empirical correlation matrix is consistent with its random matrix counterpart. Many empirical studies reveal that only a small fraction of eigenvalues and their corresponding eigenvectors contain system information while most are embedded in noises [17,52,53,54]. It has been reported that the portion of the largest eigenvalues deviating from the theoretical prediction of the counterpart random matrix is 6% [17], 4.7% [54], 2% [34], 11% [53], and 1% [35].

Furthermore, the study of [55] adds new evidence that not all eigenvalues that fall into the theoretical interval predicted by the random matrix are purely random noise but still carry some information. Derived from the eigenvector-eigenvalue identity, a study showed that dominant eigenvalues, super eigenvalues, and maximum eigenvalues could help to analyze the spectrum of the financial correlation matrix in depth [56]. In computational results and applications in financial markets, one study reviewed the previous works, including some real-world applications, and presented promising analytical techniques from random matrix theory [26,57]. Another study proposed general, exact formulas for the overlaps between the eigenvectors of large correlated random matrices with noises [58]. Besides the intro-relationship of stock markets, another study revealed a deep relationship between news and world financial indices using tools of random matrix theory [59]. Economic policy is another field that has a significant influence on the stock market, and [60] analyzed the correlation matrix and different stock network structures to reveal the implication of the correlation matrix components. The work of [61] fused previous models, which made predictions based on the arbitrarily long time horizon and introduced an ensemble of random rectangular matrices from the observations of independent Lévy processes over a fixed-time horizon. To summarize and compose a benchmark for the study of correlated time-series signals, ref. [62] used supersymmetric theory to generate the statistics of eigenvectors of the cross-correlations of correlated time-series. Another study investigated the correlations of Chinese stocks before and during the 2008 crisis based on the random matrix analysis [63].

### 2.3. Comparative Studies on Different Markets

In another thread, there are abundant studies dedicated to the comparative studies on the two major stock markets, namely the Chinese and US markets [29,64,65,66,67]. Although RMT has been applied to stock markets, there is still a lack of comprehensive studies using RMT to analyze the Chinese and US markets. How the correlations and eigenvalues behavior are related to the market switches between bull and bear markets is still not sufficiently investigated. There is a thread of literature on comparing the dynamics of markets in different countries [29,30]. Considering the signs of eigenvector components, sub-sectors of positive and negative signs can be derived from sectors in anti-correlation. The sub-sectors are detected with strong appearances in the Chinese stock market but weaker in the US stock market [44]. US and British stock exchanges are studied by using RMT on the asymmetric correlation matrix with a lag of τ [3]. One work revealed the different strengths of correlations between stocks, especially the oil sector and banking stocks in the Nigerian Stock Market (NSM) and Johannesburg Stock Exchange (JSE), for the period of 2009 to 2013, using random matrix theory [68]. Comparative analyses on two different stock markets—the S&P 500 (USA) and Nikkei 225 (JPN) via the power mapping method from the random matrix theory, and found strong consistency between the states of the two stock markets as well as the feasibility to predict critical state (market crash) [69].

Particularly, some works investigated the markets of the US and China [64,66]. According to the strong connection between financial assets and institutions and the diversity as well as the localization of the stock market, one study previously analyzed the topological structure of financial networks of two major markets of China and the US with complex network theory [29]. Several studies investigated the two markets from aspects of comovement [70], impacts of trade conflicts and pandemic [65,67,71], and conditional correlations [72]. These studies revealed the different behaviors of the two major stock markets. However, there is still a lack of comprehensive studies on the Chinese and US stock markets from the perspective of RMT. In this sense, this work aims to fill this gap by systematically investigating the two markets using correlation analysis and RMT.

## 3. Methodology

### 3.1. Construction of Correlation Matrices

For an empirical correlation matrix *C* of size N×N generated from *N* returns series of length *L*, we can construct the elements as

(2) $$C=\frac{1}{L}MM^{T},$$

where *M* is a N×L matrix with normalized return $y_{i}\left(t\right)$ for each stock at every time *t*, where

(3) $$y_{i}\left(t\right)=\frac{Y_{i}\left(t\right)-\langle Y_{i}\left(t\right)\rangle }{\sigma_{i}},$$

where $Y_{i}\left(t\right)$ stands for the return at time *t*.

The study of [73] provides a study of the eigenvalues spectrum for the Chinese stock market with a sliding window approach. The inverse participation ratio is defined as

(4) $$I^{k}=\sum_{l=1}^{N}{\left[u_{l}^{k}\right]}^{4},$$

where $u_{l}^{k}$ is the components of eigenvector $v^{k}$, to measure the deviation degree of eigenvectors [53]. A criterion of fractional Gaussian noise (fGn) is used to evaluate the autocorrelation matrix of stocks showing agreement with fGn, though the stock returns are non-Gaussian [20].

### 3.2. Eigenvalue Analysis Using RMT

RMT is a powerful tool in the analysis of eigenvalues of noisy data in various fields [15,16,74,75]. According to RMT [15,16], the eigenvalue distribution of a pure random matrix $C_{random}$ with the same size of *C* follows

(5) $$p{\left(\lambda \right)}_{random}=\frac{Q}{2\pi }\frac{\sqrt{\left(\lambda_{max}-\lambda \right)\left(\lambda -\lambda_{min}\right)}}{\lambda },$$

where $\lambda_{min}$ and $\lambda_{max}$ are the theoretical minimum and maximum eigenvalue bounds of random matrix, the *Q* is the ratio of LLNN satisfying the requirement that Q>1, L→∞, and N→∞ [17]. Using the empirical data, we can also get the empirical distribution as

(6) $$p{\left(\lambda \right)}_{random}=\frac{1}{N}\frac{dn(\lambda )}{d\lambda }.$$

Theoretically, with the knowledge of *Q*, we can determine the theoretical eigenvalue bounds as

(7) $$\lambda_{min,max}=1+\frac{1}{Q}\mp 2\sqrt{\frac{1}{Q}}.$$

With these calculations, we can construct and determine the theoretical distribution of a null hypothesis random matrix. The empirical eigenvalues that fall within the interval of $\left[\lambda_{min},\lambda_{max}\right]$ are pure random noises, and those that fall beyond the interval are the validated eigenvalues carrying true information of the system. In this way, we also get the validated corresponding eigenvectors for those validated eigenvalues. Also, we can go further to investigate the statistical validation of the eigenvectors. The distribution of the eigenvector components in $v_{i}$ for eigenvalue $\lambda_{i}$ follows the Porter-Thomas distribution [17] as

(8) $$P\left(v_{i}\right)=\frac{1}{\sqrt{2\pi }}e^{-\frac{v_{i}^{2}}{2}},$$

with which we can validate the eigenvector components by comparing the distributions. It has been reported that the distribution of eigenvector components of the largest eigenvalues shows a great difference from the theoretical predictions [17].

In short, we first construct the correlation matrix for the *N* stocks and calculate the corresponding theoretical bounds of eigenvalues predicted with RMT, and analyze the eigenvalues with special attention to the largest and second largest eigenvalues.

## 4. Data and Correlation Matrices

### 4.1. Data

In this paper, we study the stock markets of China and the United States. There are three considerations in choosing these two markets. First, both markets are major stock markets in the world with tremendous total market scales and a large number of stocks that are actively traded. Second, the two markets both experienced major market shifts between bull and bear markets demonstrating rich market dynamics and behaviors. Third, the US market and Chinese market are representatives of a much-matured market and still-developing market, respectively. We collected the daily price data of the components of the China Securities Index 300 (CSI300) and Standard & Poor’s 500 (S&P500) between 4 January 2007 and 6 November 2015. In total, the dataset covers 2149 trading days for CSI300, and 2228 trading days for S&P500. The data of CSI300 are retrieved from the CSMAR Solution Database of Shenzhen GTA Education Tech. Ltd. The data of S&P500 are extracted from Yahoo Finance service. We further selected 163 stocks from CSI300 with at least 2000 trading dates without continuous 100 non-trading dates, whereas we selected 468 stocks with at least 2100 trading dates from S&P500. Later, we refer to the screened stocks as CSI163 and S&P468, respectively [29].

### 4.2. Correlation Matrices

In a stock market, the prices of stocks fluctuate constantly showing complex behaviors. It is important to investigate the performance of individual stocks as well as the interactive behaviors among stocks. To evaluate the interactive co-movement behaviors among the prices of assets, the correlation is a fundamental concept widely used in studies of price dynamics and is used in traditional theories. When the correlation is considered, in traditional theories, like in MPT where the correlation matrices are actually inputs for the portfolio optimization [1], the correlation is assumed as fixed. Still, in the real world, the correlations fluctuate and demonstrate some collective behaviors in market crashes. As a starting point for studying the structure and behavior of markets, correlation analysis is found to be useful not only in theory but also in practices of portfolio risk estimation and optimization [33,76]. Especially during periods of crisis, highly collected co-movements of the stocks are very likely to cause significant losses for a portfolio, so it is necessary to watch the portfolio’s correlations. Also, to understand the market structure and the dynamics, it is interesting to investigate the correlations [8,33,35,77,78,79].

Following the definition and notation widely used in the literature, the Pearson correlation coefficient [8]

(9) $$\rho_{ij}=\frac{\langle Y_{i}Y_{j}\rangle -\langle Y_{i}\rangle \langle Y_{j}\rangle }{\sqrt{\left(\langle Y_{i}^{2}\rangle -{\langle Y_{i}\rangle }^{2}\right)\left(\langle Y_{j}^{2}\rangle -{\langle Y_{j}\rangle }^{2}\right)}}$$

can be calculated for each stock pair of $s_{i}$ and $s_{j}$ using the logarithmic return

(10) $$Y_{i}=\ln P_{i}\left(t\right)-\ln P_{i}\left(t-1\right).$$

The value of $\rho_{ij}$ ranges from −1 to −1, indicating a dynamic relationship for the two stocks from a complete anti-correlation to a complete correlation. For a perfect uncorrelated pair, $\rho_{ij}=0$ by definition. If there are *N* stocks in consideration, then there will be $N^{2}$ correlation coefficients fitting into a N×N correlation matrix. Correlation analysis has been applied in the study of market structures [8,28,80] and portfolio optimization [31,37,43,54].

In the RMT approach, the statistics of the eigenvalues distribution and the deviation between empirical distribution and the distribution generated from a random fashion are discussed to describe the information contribution of these deviated eigenvalues and the corresponding components of the eigenvectors. But first, the empirical results are tested against a random matrix case [31].

## 5. Eigenvalues and Eigenvectors for CSI163 and S&P468

### 5.1. Eigenvalues

**Figure 1:**
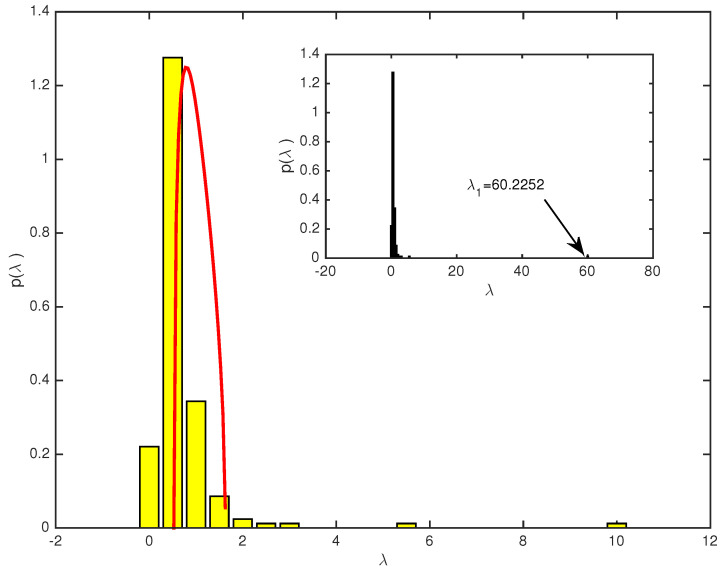
The eigenvalue distributions for CSI163 correlation matrix over the whole study period. The yellow bars are distributions of all eigenvalues calculated from the empirical correlation matrix of 163 daily log return time series, and the red curve is the theoretical distribution predicted from the random matrix theory by using a random matrix of the same size as the empirical correlation matrix. The upper bound is $\lambda_{max}=1.6267$. The inset is a plot of all empirical eigenvalues including the largest eigenvalue $\lambda_{1}=60.2252$.

**Figure 2:**
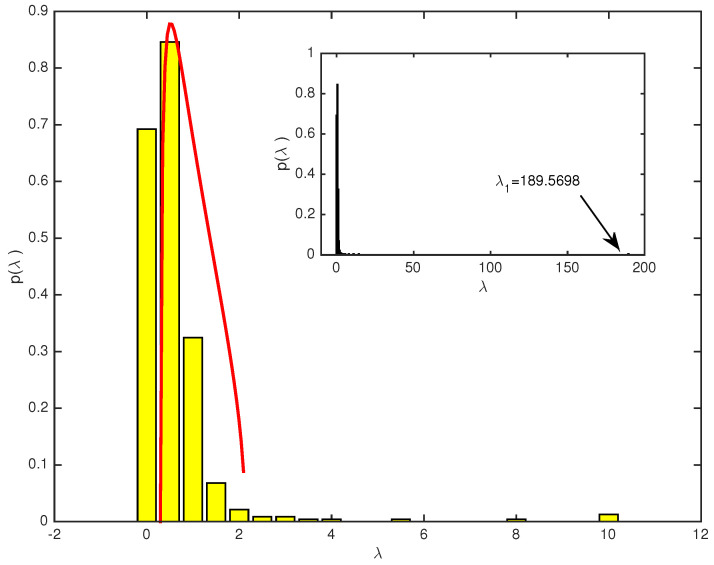
The eigenvalue distributions for S&P468 correlation matrix over the whole study period. The yellow bars are distributions of all eigenvalues calculated from the empirical correlation matrix of 468 daily logged return series, and the red curve is the theoretical distribution predicted from the random matrix theory by using a random matrix of the same size as the empirical correlation matrix. The upper bound is $\lambda_{max}=2.130$. The inset is a plot of all empirical eigenvalues, including the largest eigenvalue $\lambda_{1}=189.5698$.

Based on the correlation matrices we built in the previous section, we are ready to investigate the eigenvalues and eigenvectors of both markets. First, we use all the logged daily returns data of both two markets, CSI163 and S&P468 over the whole study period, which is 4 January 2007 and 6 November 2015 covering 2149 trading dates for the former and 2228 trading dates for the latter. We present the probability density distributions (PDF) of eigenvalues from the empirical correlation matrix and theoretically predicted by using random matrix theory for CSI163 in Figure 1 and for S&P468 in Figure 2, respectively. For both markets, we find that most empirical eigenvalues are within the RMT predicted interval with some exceptions. As shown in Figure 1, for CSI163, the theoretical predicted eigenvalues bounds are $\lambda_{min}=0.5250$ and $\lambda_{max}=1.6267$. We see that there are 7 eigenvalues are larger than the largest eigenvalue predicted by RMT, i.e., 4.29% of all eigenvalues fall beyond the interval. The largest eigenvalue $\lambda_{1}=60.2252$ is nearly 37 times the predicted largest eigenvalue, i.e., λ1λ1λmaxλmax=37.0238. For S&P468, as shown in Figure 2, the largest eigenvalue $\lambda_{1}=189.5698$ which is almost 89 times the bound predicted by RMT. There are 12 eigenvalues that are larger than the bound, i.e., 3.56% are beyond the interval and carry real market information.

**Figure 3:**
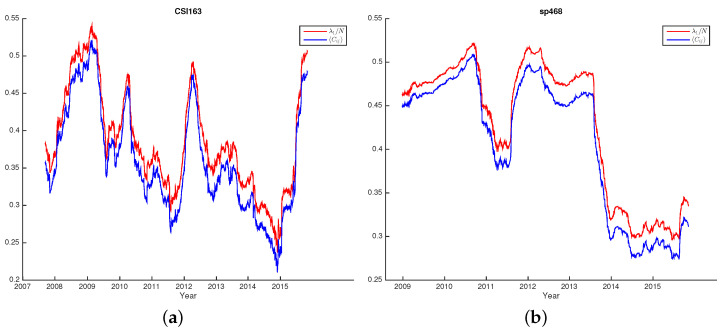
The largest eigenvalue $\lambda_{1}/N$ and the average correlation $\langle C_{ij}\rangle$ for all sliding windows of CSI163 (**a**) and S&P468 (**b**). We see that the two curves fit very well.

Using the sliding window approach, we can investigate the dynamic properties of eigenvalue distributions. For CSI163 and S&P468, we use the window size $L_{csi163}=170$ and $L_{S\&P468}=500$, respectively, to satisfy the requirement of Q=L/N>1. In choosing the window sizes, basically, we desire a window that is large enough to cover significant market periods. A shorter window might lead to short-term noises that do not reflect the fundamental dynamics of the markets. Furthermore, the window moves at a step of one trading date; this allows our sliding windows to move smoothly with the finest possible granularity and capture detailed market behaviors. For each sliding window, we use the data of *N* stocks to calculate the pairwise correlation matrix *C*, from which we further calculate the $\lambda_{1}/N$ and average correlation $\langle C_{ij}\rangle =\sum C_{ij}/N^{2}$. As shown in Figure 3a,b, we see that for both markets, the values of $\lambda_{1}/N$ and the average correlation $\langle C_{ij}\rangle$ correlated very well over the whole study period indicating that $\lambda_{1}/N$ is a good estimator of the average correlation $\langle C_{ij}\rangle$ as we have introduced previously.

**Figure 4:**
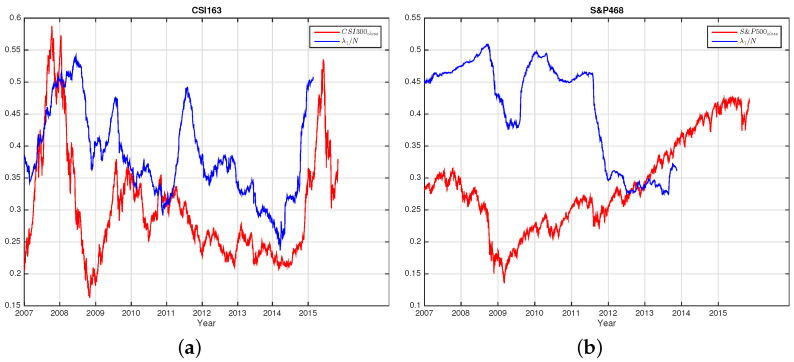
The largest eigenvalue $\lambda_{1}/N$ and the index close price of CSI300 (**a**) and S&P500 (**b**). The largest eigenvalue $\lambda_{1}/N$ curves are left shifted 170 trading dates for CSI300 and 500 trading dates for S&P500, for the window size is 170 for CSI163 and 500 for S&P468. For better visualizations, we shrink the indices of CSI300 and S&P500 10,000 times and 5000 times, respectively. We see that the shifted curves of $\lambda_{1}/N$ are similar to the indices.

In Figure 4a,b, we plot the largest eigenvalue $\lambda_{1}/N$ and the index close prices of CSI300 (a) and S&P500 (b). After the left shifting, we find that $\lambda_{1}/N$ and the index itself show similar trends. This shows that $\lambda_{1}/N$ is also an indicator of the index itself. For CSI163, the trend similarity is relatively more obvious than that of S&P468. If we do not perform left shifts, we find that $\lambda_{1}/N$ is anti-co-move with the index showing that during market crashes, the $\lambda_{1}/N$ (also the average correlation $\langle C_{ij}\rangle$) becomes larger, i.e., the stocks of the market are correlated, whereas during calm periods, the $\lambda_{1}/N$ becomes small indicating fewer correlations among stocks.

**Figure 5:**
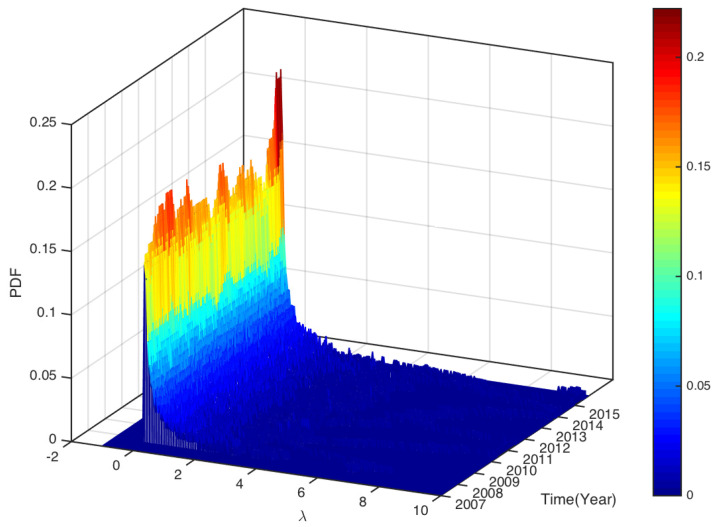
The PDF of all eigenvalues (excluding the largest eigenvalue $\lambda_{1}$) distribution for all sliding windows over the study period of CSI163.

**Figure 6:**
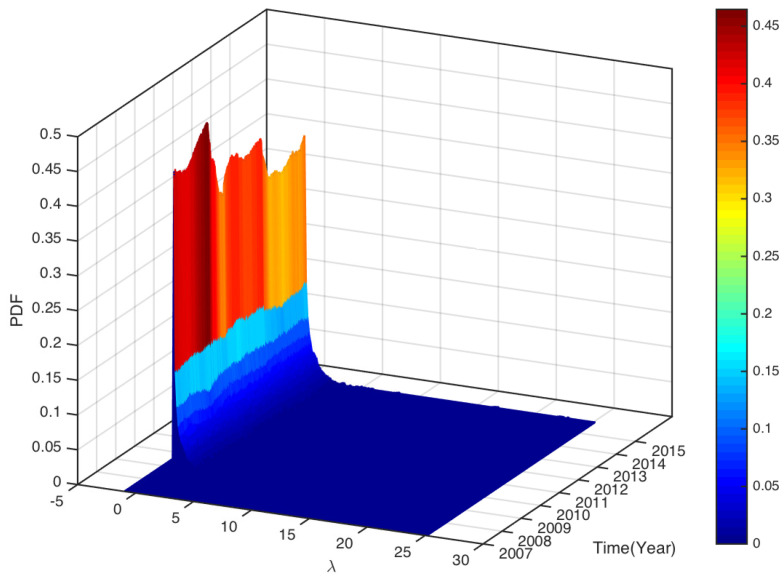
The PDF of all eigenvalues (excluding the largest eigenvalue $\lambda_{1}$) distribution for all sliding windows over the study period of S&P468.

**Figure 7:**
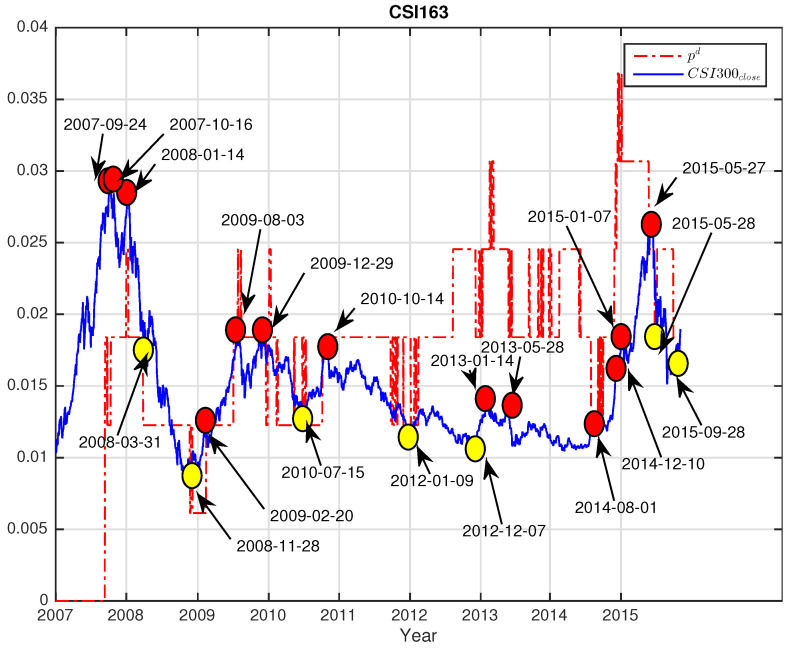
The fraction $p^{d}$ of eigenvalues beyond the predicted largest eigenvalue versus the index close price for CSI163 over the study period. For better visualization, we rescale the index values by shrinking 200,000 times. The coincidences of changes of fraction $p^{d}$ and the index closing price are marked out in red dots for local maximums and yellow dots for local minimums on the price curve with dates.

**Figure 8:**
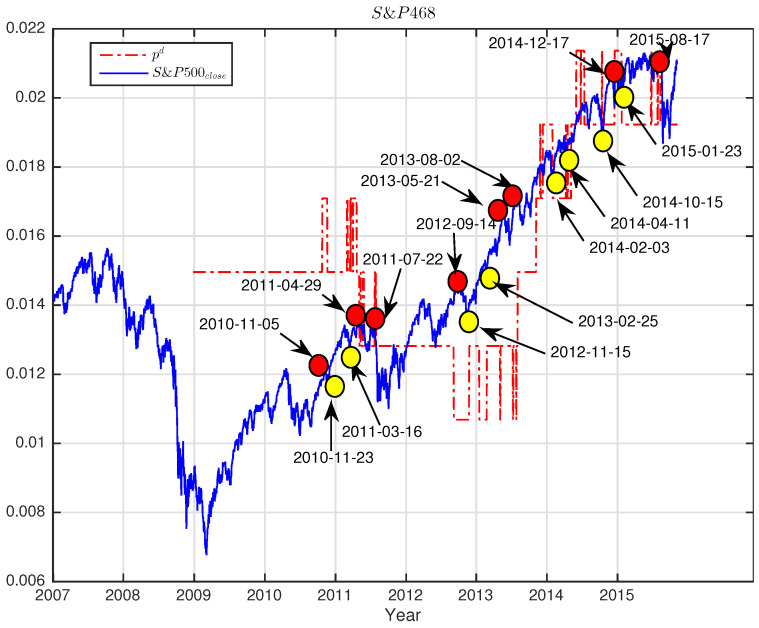
The fraction $p^{d}$ of eigenvalues beyond the predicted largest eigenvalue versus the index close price for S&P468 over the study period. For better visualization, we rescale the index values by shrinking 100,000 times. The coincidences of changes of fraction $p^{d}$ and the index closing price are marked out in red dots for local maximums and yellow dots for local minimums on the price curve with dates.

To see how the eigenvalues distributed in the whole study period. In Figure 5 and Figure 6, we plot the distributions of the eigenvalues (excluding the largest eigenvalue) of all sliding windows over the study periods CSI163 and S&P468, respectively. As the figures show, most eigenvalues are very small. Though many eigenvalues are within the bounds of prediction based on RMT, we also observe some eigenvalues are larger than the upper bound $\lambda_{max}=3.9172$ for CSI163 and $\lambda_{max}=3.8709$ for S&P468. We define the fraction of eigenvalues that are larger than the predicted $\lambda_{max}$ using RMT as

(11) $$p^{d}=\frac{\left|\lambda >\lambda_{max}\right|}{N},$$

i.e., the ratio of the number of eigenvalues deviated beyond $\lambda_{max}$ to the total number of eigenvalues *N*. Since the eigenvalues carry meaningful information about the market, this ratio can be employed as an indicator describing how much information is embedded in the distribution of the empirical eigenvalues. Using the sliding window approach, we calculate the fraction for each window and plot with the index close price for CSI163 in Figure 7 and S&P468 in Figure 8, respectively.

For better visualizations, we shrink the index values of 200,000 times for $CSI163_{close}$ and 100,000 times for $S\&P468_{close}$, respectively. As we can see, the values of $p^{d}$ stay unchanged between sudden changes, so the curves of $p^{d}$ show a shape of discrete stages with ups and downs. More interestingly, we find that the changes of $p^{d}$ coincide with the changes in index closing prices. As shown in Figure 7 for CSI163 and Figure 8 for S&P468, the changing points of the $p^{d}$ precisely mark out the local minimums (marked with yellow dots) and local maximums (marked with red dots) of the index itself.

We see that $p^{d}$ is relatively stable with many fixed periods, but the changes of $p^{d}$ can match with the significant market changes in index closing prices. Some of them are even leading the index for several days. This observation indicates that $p^{d}$ has the potential to monitor the market situation. Once the $p^{d}$ changes value, investors must be cautious and pay particular attention to the market fluctuations both of surges and crashes. This information might also be useful in designing trading strategies to catch major market mode switches.

In Table 1, we summarize the properties of eigenvalues that deviate beyond the $\lambda_{max}$. We see that only a very small fraction of eigenvalues is larger than the theoretically predicted eigenvalue. On average, only 3.0268 eigenvalues for CSI163 and 7.2250 eigenvalues for S&P468 are beyond the bounds. The average fraction is $\langle p^{d}\rangle =0.0186$ for CSI163 and $\langle p^{d}\rangle =0.0154$ for S&P468, respectively.

### 5.2. Largest Eigenvalue

**Figure 9:**
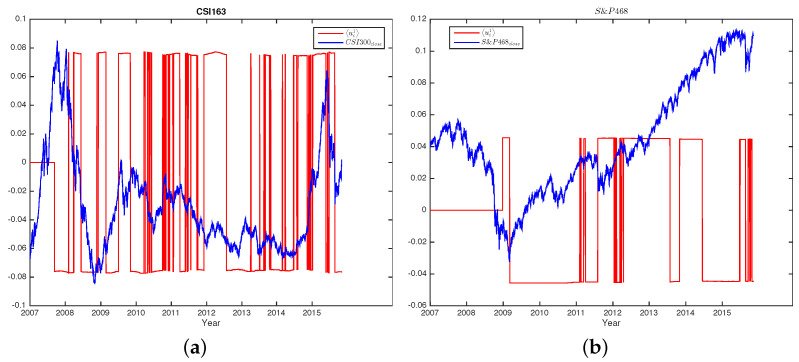
The average of eigenvector components corresponding to the largest eigenvalue $\langle u_{i}^{1}\rangle$ and the index close price of both CSI300 (**a**) and S&P500 (**b**). For better visualizations, we shrink the index close price by 25,000 times and 10,000 times for CSI300 and S&P500, respectively. We see that the changes of $\langle u_{i}^{1}\rangle$ happen on the dates when the markets change.

To study the eigenvector $u^{1}$ corresponding to the largest eigenvalue $\lambda_{1}$, we take an average of all eigenvector components. Since the $\lambda_{1}$ stands for the whole market, we expect that the average components are related to the index. We plot the $\langle u_{i}^{1}\rangle$ with the index close prices of both markets for each sliding window in Figure 9a,b. As shown in the figures, the value of $\langle u_{i}^{1}\rangle$ changes happened on the dates or periods of major market changes. For the eigenvector $u^{1}$, we also confirm that all components have the same sign, either positive or negative [81], i.e., all stocks contribute to the movement of the market on the eigenvector $u^{1}$ in the same direction; they either climb or fall. It is worth noting that, in practice, one might choose to remove the market mode of the largest eigenvalue before analyzing the eigenvalues. Here, we directly analyze the second-largest eigenvalue and the corresponding eigenvector for simplicity.

### 5.3. Second Largest Eigenvalue

It is believed that the largest eigenvalue $\lambda_{1}$ stands for the market mode itself, whereas the second largest $\lambda_{2}$ eigenvalue and its corresponding eigenvector $u^{2}$ contain more information about the market. Now, we focus on the distribution of the components in $u^{2}$. As we know, the values of components in eigenvectors represent the weights for the corresponding eigenvector; the best idea to allocate investment in portfolio management is that we long the assets with positive signs and short the assets with negative signs. The eigenportfolio based on eigenvector $u^{j}$ is given as:

(12) $$P_{j}=\sum_{i=1}^{N}\frac{1}{\sqrt{\lambda_{j}}}\frac{u_{i}^{j}}{\sigma_{i}}Y_{i},$$

where *N* is the number of assets, $U_{i}^{j}$ is the component for asset $s_{i}$ in eigenvector $u_{j}$, and $Y_{i}$ is the return for asset $s_{i}$. This indicates that larger eigenvalues $\lambda_{i}$ bring fewer weights for assets in a risky portfolio, whereas smaller eigenvalues bring smaller risks with greater weights on the assets.

For industry $I_{i}$, the contribution of $I_{i}$ is defined as

(13) $$I_{i}^{j}\left(t\right)=\sum_{k\in I_{i}}{\left(u_{k}^{j}\right)}^{2},$$

where $u_{k}^{j}$ is the value of the stock belonging to industry $I_{i}$. By dividing over the total values of all industries, we get the normalized contribution for industry $I_{i}$

(14) $${\bar{I}}_{i}^{j}\left(t\right)=\frac{I_{i}^{j}\left(t\right)}{\sum_{i}I_{i}^{j}\left(t\right)}.$$

Compared with another approach [73], the normalized values allow comparison between any two industries, thus making the ranking of industries possible. Of course, Equation (14) also indicates that $\sum_{i}{\bar{I}}_{i}^{j}\left(t\right)=1$.

**Figure 10:**
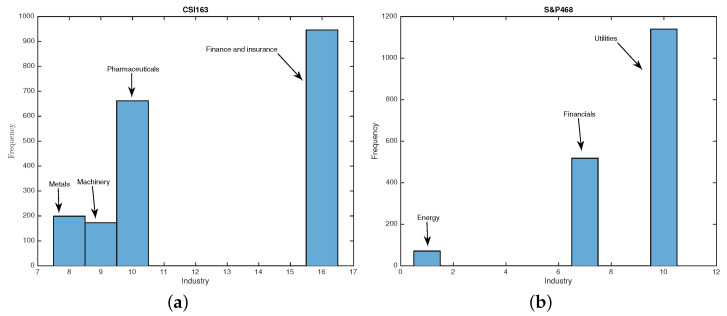
The frequencies of industries appearing in the largest values of eigenvector components of CSI163 (**a**) and S&P468 (**b**). For CSI163, four industries appear in the largest eigenvector components, whereas there are three industries for that of S&P468.

Using Equations (13) and (14), we calculate and rank all industries in all sliding windows for both CSI163 and S&P468. For a given date, we can get the contributions from all sectors to the eigenvector components for the second largest eigenvalue $u^{2}$. We investigate which industries appear in the components with the largest values. In Figure 10a,b, we plot the histograms for industries that appeared for CSI163 and S&P468, respectively. We find that four industries appeared for CSI163, which are finance and insurance, pharmaceuticals, machinery, and metals, whereas for S&P468, we find only three industries appeared, which are utilities, financials, and energy. This reveals the leading industrial sectors for the two markets over the whole study period.

### 5.4. Market Switching

Both the Chinese and US markets experienced significant fluctuations during our study period covering some major market mode changes of bull markets and bear markets. In our study period between 4 January 2007 and 6 November 2015, the Chinese stock market enjoyed a bull market period from 2007 to 2008 and surged to its historical height in 2008 but soon suffered a major crash and only partially recovered in the middle of 2008 and stepped into bear market mode before long. This bear market mode lasted for almost seven years, and only finished in 2015, being replaced by a rocket bull market mode. Unfortunately, the 2015 bull market was very short and tumbled greatly into bear market mode again with huge drops. For S&P500, the US stock markets also suffered a great market crisis in 2008, but the market changed into a very long climbing bull market in 2009.

To investigate how the $u^{2}$ changes before and after a market crash, we choose a case study period between 24 July 2008 and 16 February 2009 for CSI300 centering with a market turning point on 4 November 2008, covering 135 trading days and a period between 26 December 2008 and 2 June 2009, and for S&P500 centering with a market turning point on 9 March 2009, covering 108 trading days. We denote the ranking for stock $s_{i}$ at time *t* as $R_{i}\left(t\right)$ according to the normalized values. For a period of $\left[t_{s},t_{e}\right]$ of length $L_{s,e}$, the averaged ranking for $s_{i}$ is

(15) $$\langle R_{i}\left(t\right)\rangle =\frac{1}{L_{s,e}}\sum_{t=t_{s}}^{t_{e}}R_{i}\left(t\right),$$

where $L_{s,e}=t_{e}-t_{s}+1$ is the number of trading dates in the period. By calculating all averaged rankings for all of the stocks in both periods before and after the market crash, we can get the top and bottom 10 stocks for CSI163 and S&P468. The top and bottom 10 stocks according to the averaged ranking for CSI163 in the *Fall* stage and *Climb* stage are presented in Table 2 and Table 3, respectively. The same lists are presented in Table 4 and Table 5 for the Fall and Climb stages of S&P468.

The tables reveal some exciting results. In Table 2, we see that stocks of finance and real estate occupy the bottom ten while stocks of pharmaceuticals dominate the top 10 in the Fall stage of CSI300, and this phenomenon remains unchanged during the Climbing stage after the market turning point. This indicates and confirms again that financials are not the only dominating players in the Chinese stock market. In the Climbing stage, as shown in Table 2, stocks of pharmaceuticals still dominate the top 10, and the stocks of finance remain at the bottom part. This shows that the internal structure of the CSI300 market remains almost unchanged before and after the market crashes.

Being opposite to CSI163, S&P468 demonstrates a different behavior before and after the crash period. As shown in Table 4, stocks of financials dominate the top positions with the smallest rankings; in other words, stocks of financials play significant roles in the Fall stage; however, stocks of energy collectively occupy the bottom 10. When the market entered the Climb stage, passing the turning point, the whole rankings reversed with stocks of energy becoming the top stocks whereas the financials stocks fell to the bottom, as shown in Table 5.

## 6. Conclusions and Discussion

In this study, we applied random matrix theory to study the eigenvalues and their eigenvectors of the US and Chinese stock markets. The correlation properties are studied, and some eigenvalues of the correlation matrices beyond the predicted bounds are observed in both markets. The largest eigenvalues $\lambda_{1}$ are dozens of times larger than the predicted $\lambda_{max}$. They are found to be potential market indicators. Eigenvalue deviation fractions beyond the predicted largest eigenvalue are observed to pinpoint market turning points. For the two markets, the most influential industry sectors are identified. They behave differently when the market crashes. These findings provide information on the dynamics of eigenvalues and eigenvectors. This is useful for investors and regulators to monitor the markets. On the other hand, the eigenvalues are related to factor models. The largest eigenvalue stands for the market itself and the corresponding eigenvector has impacts on most stocks, described as the single factor model for stock $s_{i}$: $r_{i}=\beta_{i}r_{M}+e_{i}$, where $r_{M}$ is the market return, for *N* stocks, the correlation matrix has one dominant eigenvalue. The CAMP is a special case of a single factor model. However, other eigenvalues are beyond the predicted $\lambda_{max}$. It is natural to model the returns in multi-factors as proposed in arbitrage pricing theory (APT), $r_{i}=\sum \beta_{ki}f_{k}+e_{i}$, where $f_{k}$ is the *k*th factor. Since the eigenvalues embedded in the predicted bounds represent noises, it is natural to choose the top *k* largest eigenvalues $\lambda_{max-k},\cdots ,\lambda_{max-1}$; thus, we get *k* corresponding eigenvectors $v_{max-k},\cdots ,v_{max-1}$. In other words, the *k* principle components in the PCA. To simplify the model, it is reasonable to consider the sector information revealed in the eigenvectors; in other words, the corresponding eigenvector components belonging the the sector are reserved.

Last but not least, the present work still has several limitations that should not be neglected and are worth further efforts in future works. First, this work only considered two major markets in an outdated time period. More global markets and updated periods can be considered in future work. Second, this work provides findings largely through empirical analysis rather than rigorous statistical approaches. In order to further validate the findings, statistical testing should be considered. Third, this work only reports observations, and no practical applications are developed to further evaluate the values of the methodology and findings. In the future, applications like quantitative trading strategies, portfolio management, and risk management can be developed around the findings to demonstrate the values in financial practices.

## Figures and Tables

**Table 1:** Properties of eigenvalue deviation fraction $p^{d}$ for CSI163 and S&P468. The avg. number is the average number of eigenvalues deviated beyond the predicted upper bounds $\lambda_{max}$.

Market	Avg. Number	$p_{\min}^{d}$	$p_{\max}^{d}$	$\langle p^{d}\rangle$
CSI163	3.0268	0.0061	0.0368	0.0186
S&P468	7.2250	0.0107	0.0214	0.0154

**Table 2:** The top ten and bottom ten stocks of the second largest eigenvalue $u^{2}$ of CSI163 ranked by the average $u^{2}$ components values in the Fall stage between 24 July 2008 and 4 November 2008.

Top 10			
**Rank**	**Tick**	**Stock Name**	**Industry**
1	2007	Hualan Biological Engineering Inc.	Pharmaceuticals
2	600,867	Star Lake Bioscience Co., Inc.	Pharmaceuticals
3	600,085	Beijing Tongrentang Co., Ltd.	Pharmaceuticals
4	963	Huadong Medicine Co., Ltd.	Wholesale
5	600,332	Sichuan Hongda Co., Ltd.	Metals
6	600,108	Gansu Yasheng Industrial (Group) Co., Ltd.	Agriculture
7	600,535	Nanjing Chixia Development Co., Ltd.	Real estate
8	600,277	Jiangsu Hengrui Medicine Co., Ltd.	Pharmaceuticals
9	600,089	TBEA Co., Ltd.	Machinery
10	999	Sanjiu Medical & Pharmaceutical Co., Ltd.	Pharmaceuticals
**Bottom 10**			
**Rank**	**Tick**	**Stock Name**	**Industry**
154	46	Oceanwide Construction Group Co., Ltd.	Real estate
155	601,988	China Construction Bank	Finance
156	2	China Vanke Co., Ltd.	Real estate
157	600,048	Poly Real Estate Group Co., Ltd.	Real estate
158	601,398	Guangshen Railway	Transportation
159	600,016	China Minsheng Banking Corp. Ltd.	Finance
160	600,015	Hua Xia Bank Co., Ltd.	Finance
161	1	Shenzhen Development Bank Co., Ltd.	Finance
162	600,036	China Merchants Bank Co., Ltd.	Finance
163	600,000	Shanghai Pudong Development Bank	Finance

**Table 3:** The top ten and bottom ten stocks of the second largest eigenvalue $u^{2}$ of CSI163 ranked by the average $u^{2}$ components values in the Climb stage between 4 November 2008 and 16 February 2009.

Top 10			
**Rank**	**Tick**	**Stock Name**	**Industry**
1	999	Sanjiu Medical & Pharmaceutical Co., Ltd.	Pharmaceuticals
2	2007	Hualan Biological Engineering Inc.	Pharmaceuticals
3	629	Panzhihua New Steel & Vanadium Co., Ltd.	Metals
4	600,089	TBEA Co., Ltd.	Machinery
5	600,085	Beijing Tongrentang Co., Ltd.	Pharmaceuticals
6	538	Yunnan Baiyao Industry Co., Ltd.	Pharmaceuticals
7	963	Huadong Medicine Co., Ltd.	Wholesale
8	729	Beijing Yanjing Brewery Co., Ltd.	Food & Beverage
9	600,535	Nanjing Chixia Development Co., Ltd.	Real estate
10	600,332	Sichuan Hongda Co., Ltd.	Metals
**Bottom 10**			
**Rank**	**Tick**	**Stock Name**	**Industry**
459	157	Changsha Zoomlion Heavy Industry	Machinery
460	600,030	CITIC Securities Co., Ltd.	Finance
461	600,585	Jiangsu Changjiang Electronics Technology	Electronics
462	601,988	China Construction Bank	Finance
463	601,398	Guangshen Railway	Transportation
464	1	Shenzhen Development Bank Co., Ltd.	Finance
465	600,015	Hua Xia Bank Co., Ltd.	Finance
466	600,016	China Minsheng Banking Corp. Ltd.	Finance
467	600,036	China Merchants Bank Co., Ltd.	Finance
468	600,000	Shanghai Pudong Development Bank Co., Ltd.	Finance

**Table 4:** The top ten and bottom ten stocks of the second largest eigenvalue $u^{2}$ of S&P468 ranked by the average $u^{2}$ components values in the Fall stage between 26 December 2008 and 9 March 2009.

Top 10			
**Rank**	**Tick**	**Stock Name**	**Industry**
1	STI	SunTrust Banks	Financials
2	ZION	Zions Bancorp	Financials
3	MTB	M&T Bank Corp.	Financials
4	CMA	Comerica Inc.	Financials
5	WFC	Wells Fargo	Financials
6	BBT	BB&T Corporation	Financials
7	JPM	JPMorgan Chase & Co.	Financials
8	RF	Regions Financial Corp.	Financials
9	LEN	Lennar Corp.	Consumer Discretionary
10	PNC	PNC Financial Services	Financials
**Bottom 10**			
**Rank**	**Tick**	**Stock Name**	**Industry**
459	EOG	EOG Resources	Energy
460	MUR	Murphy Oil	Energy
461	OXY	Occidental Petroleum	Energy
462	HP	Helmerich & Payne	Energy
463	NBL	Noble Energy Inc.	Energy
464	XEC	Cimarex Energy	Energy
465	APC	Anadarko Petroleum Corp.	Energy
466	DO	Diamond Offshore Drilling	Energy
467	DVN	Devon Energy Corp.	Energy
468	APA	Apache Corporation	Energy

**Table 5:** The top ten and bottom ten stocks of the second largest eigenvalue $u^{2}$ of S&P468 ranked by the average $u^{2}$ components values in the Climb stage between 10 March 2009 and 2 June 2009.

Top 10			
**Rank**	**Tick**	**Stock Name**	**Industry**
1	APA	Apache Corporation	Energy
2	DVN	Devon Energy Corp.	Energy
3	ETR	Entergy Corp.	Utilities
4	DO	Diamond Offshore Drilling	Energy
5	NBL	Noble Energy Inc.	Energy
6	APC	Anadarko Petroleum Corp.	Energy
7	FE	FirstEnergy Corp.	Utilities
8	OXY	Occidental Petroleum	Energy
9	MUR	Murphy Oil	Energy
10	XOM	Exxon Mobil Corp.	Energy
**Bottom 10**			
**Rank**	**Tick**	**Stock Name**	**Industry**
459	USB	US Bancorp	Financials
460	JPM	JPMorgan Chase & Co.	Financials
461	RF	Regions Financial Corp.	Financials
462	WFC	Wells Fargo	Financials
463	BBT	BB&T Corporation	Financials
464	PNC	PNC Financial Services	Financials
465	ZION	Zions Bancorp	Financials
466	CMA	Comerica Inc.	Financials
467	MTB	M&T Bank Corp.	Financials
468	STI	SunTrust Banks	Financials

## Data Availability

All data used in this study are publicly available from financial websites.
